# Differential effects of food availability on minimum and maximum rates of metabolism

**DOI:** 10.1098/rsbl.2016.0586

**Published:** 2016-10

**Authors:** Sonya K. Auer, Karine Salin, Agata M. Rudolf, Graeme J. Anderson, Neil B. Metcalfe

**Affiliations:** 1 Institute of Biodiversity, Animal Health and Comparative Medicine, University of Glasgow, Glasgow, UK; 2 Institute of Environmental Sciences, Jagiellonian University, Krakow, Poland

**Keywords:** energy metabolism, metabolic power, plasticity, *Salmo trutta*, standard metabolic rate

## Abstract

Metabolic rates reflect the energetic cost of living but exhibit remarkable variation among conspecifics, partly as a result of the constraints imposed by environmental conditions. Metabolic rates are sensitive to changes in temperature and oxygen availability, but effects of food availability, particularly on maximum metabolic rates, are not well understood. Here, we show in brown trout (*Salmo trutta*) that maximum metabolic rates are immutable but minimum metabolic rates increase as a positive function of food availability. As a result, aerobic scope (i.e. the capacity to elevate metabolism above baseline requirements) declines as food availability increases. These differential changes in metabolic rates likely have important consequences for how organisms partition available metabolic power to different functions under the constraints imposed by food availability.

## 1. Introduction

Whether migrating thousands of kilometres or diving towards the deep ocean bottom, animals are capable of accomplishing remarkable aerobic feats. Not all organisms, however, are endowed with such high metabolic power. Maximum rates of aerobic metabolism are typically reached during or following exhaustive exercise but can differ by up to threefold among individuals [1] and greater than an order of magnitude across species [2]. The origins and scope of this diversity are due in part to the energy demands associated with the different ecological roles of organisms (e.g. [2,3]), but environmental conditions can also constrain metabolic processes and contribute to observed variation. Aerobic metabolism uses oxygen to convert food into more usable forms of energy and, as such, is sensitive to both acute and long-term changes in both temperature and oxygen availability [4–6]. However, we know little about whether maximum metabolic rates are affected by food availability.

Changes in maximum metabolic rate (MMR) may have important consequences for an organism's ability to cope with variable food conditions. This is because MMR not only defines the upper boundary to aerobic capacity, but together with standard metabolic rate (SMR, the minimum oxygen consumption required to maintain homeostasis) also determines an organism's aerobic scope (AS). AS is the absolute difference between maximum and SMR and is a measure of the degree to which metabolism can be increased above baseline requirements to finance important functions such as digestion, locomotion, growth and reproduction. SMR is thought to reflect the cost of maintaining the metabolic machinery needed to finance MMR [7,8] and is known to be a flexible trait, typically changing as an increasing function of food availability [9,10]. As such, food-induced shifts in SMR may also affect MMR in parallel. However, if MMR does not increase with food intake then AS would be expected to become more constrained at higher food availabilities, but this remains untested. Here, we use a food manipulation experiment to examine flexibility in the standard and maximum metabolic rates of juvenile brown trout (*Salmo trutta*) in response to food availability and its consequences for AS.

## 2. Material and methods

Juvenile wild-caught brown trout were collected and brought into the laboratory, housed in individual compartments in a flow-through stream system, and fed *ad libitum* for three months while they acclimated to the temperature controlled room (11.5 ± 0.5°C) and its 12 L : 12 D cycle. Fish (*n* = 116) were then placed on an intermediate food ration (see below). After 28 days, their standard and maximum metabolic rates were measured. At this time, the fish ranged in body mass from 5.37 to 12.67 g (mean ± 1 s.e.: 8.45 ± 0.13 g). The fish were then placed on one of three rations: one-third of the fish remained on the intermediate ration, while the other two-thirds were placed on either a low or an *ad libitum* ration until their metabolic rates were measured again 28 days later. Rations (in calories) for each fish were calculated as a function of its body mass (*W*, g) and the water temperature (*T*, °C) for the three food levels as follows: low food = 2.04 *W*^0.73^e^(0.10*T*)^, intermediate food = 2.91 *W*^0.737^e^(0.154*T*)^ and *ad libitum* food = 4.29 *W*^0.767^e^(0.21*T*)^ [11]. The daily ration (mg trout pellets) for each fish was then determined by converting the required daily caloric intake into trout pellets (mg) using values for the energetic content of the trout pellets (Inicio Plus from BioMar Ltd, Grangemouth, UK). Body mass was measured half way in between respirometry trials to adjust food rations as the fish grew.

Both SMR and MMR were measured at 11.5°C. Fish were fasted for 48 h prior to measurement of their SMR to ensure that the additional metabolic costs of digestion did not inflate estimates of their baseline oxygen consumption [12–14]. SMR was measured over a 20 h period using continuous flow-through respirometry. Water flowed through the glass respirometry chambers (400 ml volume) at 1.47 l h^−1^ for the first metabolic measurement and then at 1.68 l h^−1^ for the second measurement 28 days later to accommodate fish growth. These flow rates ensured that oxygen consumption rates were detectable but oxygen levels remained above 80% saturation. SMR was calculated by taking the mean of the lowest 10th percentile of oxygen consumption measurements after excluding the lower outliers, i.e. those measurements below 2 s.d. from this mean [1,10]. MMR was then determined using an exhaustive chase protocol followed immediately by measurement of peak excess post-exercise oxygen consumption using closed-system respirometry [1,10]. Briefly, each fish was chased to exhaustion (less than 2 min) against a circular current (600 l h^−1^) in a bucket. Exhaustion was determined when a fish could no longer swim and was unresponsive when picked up by hand. It was then transferred immediately (less than 10 s) to a glass respirometry chamber (400 ml volume) in a closed system where water circulated at 7.35 l h^−1^ by way of a peristaltic pump. Values of SMR and MMR for each fish were then used to calculate their AS (AS = MMR − SMR).

We used the PROC MIXED procedure in SAS v. 9.3 (SAS Institute, Cary, NC, USA) to test whether metabolic rates changed with food availability. Metabolic rates and body mass were log_10_-transformed prior to analyses. Each model included metabolic rate as the dependent variable, food level and measurement time and their interaction as categorical predictors, and fish identity as a random effect to control for repeated measures. When the interaction between food regime and measurement time was statistically significant, changes in metabolism were further evaluated by testing whether the final metabolic measurement was significantly different from the initial measurement for each food regime. There was heterogeneity in the slopes of SMR versus body mass within but not across measurements, so we used the model explained above to examine changes in mass-independent SMR by using the residuals of each metabolic trait as a function of the body mass of all fish across both measurements. All data are available in the Dryad Digital Repository [15].

## 3. Results

**Figure 1.**
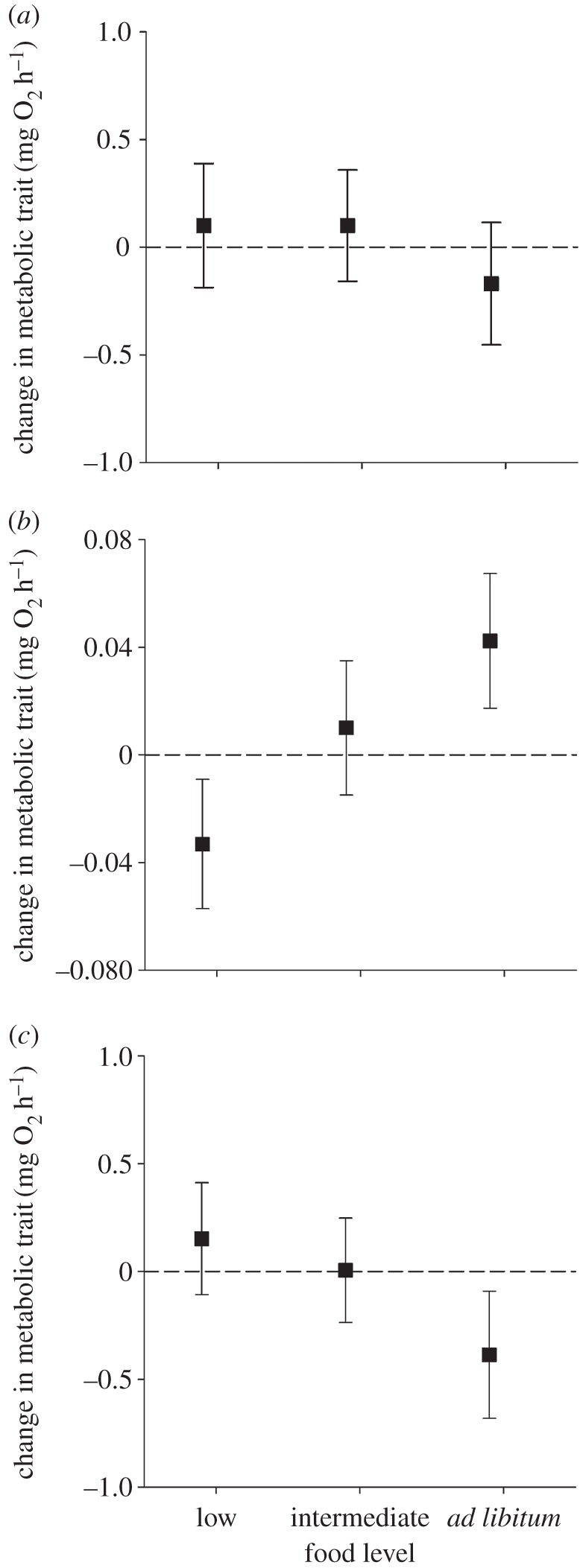
Change in maximum metabolic rate (MMR), standard metabolic rate (SMR), and aerobic scope (AS) of juvenile brown trout as a function of changing food availability. SMR and MMR were first measured after fish had been on an intermediate ration for 28 days and then again after they had been switched to either a lower, intermediate (i.e. the same as previously), or higher *ad libitum* ration for an additional 28 days. AS is defined as the difference between SMR and MMR for each fish. Plotted are back-transformed metabolic rate values (± 95% CI) standardized for a 10 g fish; positive/negative values indicate an increase/decrease in metabolic rate relative to initial values. Change in maximum metabolic rate (MMR), standard metabolic rate (SMR), and aerobic scope (AS) of juvenile brown trout as a function of changing food availability. SMR and MMR were first measured after fish had been on an intermediate ration for 28 days and then again after they had been switched to either a lower, intermediate (i.e. the same as previously), or higher *ad libitum* ration for an additional 28 days. AS was defined as the difference between SMR and MMR for each fish. Plotted are back-transformed metabolic rate values (± 95% CI) standardized for a 10 g fish; positive/negative values indicate an increase/decrease in metabolic rate relative to initial values.

MMR did not change in response to food availability (table 1 and figure 1). However, SMR changed over that same treatment period, with the direction of change differing between food levels (table 1 and figure 1). Fish decreased their SMR when switched to the lower food level (*t*_113_ = −2.66, *p* = 0.009), did not change their SMR when kept on the same intermediate ration (*t*_113_ = 1.22, *p* = 0.23), and increased their SMR when switched to the higher *ad libitum* rations (*t*_113_ = 3.27, *p* = 0.001). As a result of these shifts in SMR but not MMR, AS was also influenced by food level (table 1 and figure 1): it was unaltered in fish switched to the lower food level (*t*_113_ = 1.23, *p* = 0.223) or kept on the same intermediate ration (*t*_113_ = 0.17, *p* = 0.869), but decreased in fish switched to *ad libitum* rations (*t*_113_ = −2.14, *p* = 0.034).

**Table 1.** Results from linear models testing the effects of food level and measurement time on the maximum metabolic rate (MMR), standard metabolic rate (SMR), and aerobic scope (AS) measured before and after juvenile brown trout (*Salmo trutta*) had been kept on low, intermediate or *ad libitum* rations for one month.

trait	parameter	*F*	d.f.	*p*-value
MMR	food	0.29	2,113	0.746
	time	0.08	1,112	0.782
	food × time	1.50	2,112	0.227
	body mass	349.80	1,112	<0.001
SMR	food	2.83	2,113	0.063
	time	1.18	1,113	0.279
	food × time	9.08	2,113	<0.001
AS	food	1.04	2,113	0.357
	time	0.37	1,112	0.545
	food × time	4.24	2,112	0.017
	body mass	245.18	1,112	<0.001

## 4. Discussion

The key result from our study is that SMR changed in response to food intake without any corresponding shift in MMR. This differential sensitivity among the two components of metabolism is contrary to the hypothesis that SMR reflects the idling cost of the metabolic machinery needed to finance MMR [7,8] and, by extension, its prediction that changes in SMR should lead to corresponding changes in MMR. Rather, contrasting responses of SMR and MMR suggest that they are controlled by different underlying processes. Food intake is known to affect the masses of organs such as the viscera that contribute to whole organism SMR [16]. By contrast, skeletal muscle is thought to be the predominant contributor to MMR [17], but tends to decrease in mass only in response to prolonged starvation [18]. Thus, MMR may change in response to these more extreme conditions and warrants further attention.

Differential changes among SMR and MMR may, in turn, affect metabolic budgeting decisions because of their consequences for AS. AS represents the overall capacity to fuel functions above baseline energy expenditure and is known to have a positive effect on feeding capacity [19], locomotor ability [20] and growth rate [1]. However, animals cannot meet the aerobic demands of all of these functions simultaneously, so trade-offs can arise [21]. For example, swimming ability can be sacrificed to prioritize the allocation of aerobic power to digestion [22–24]. A decrease in AS with increasing food levels may therefore represent an increasing constraint on the organism [21]. However, it is important to note that the effect of food availability on AS that we observed is driven entirely by a change in SMR which itself can also have positive effects on growth rate [10] as well as digestion efficiency [25]. As such, the overall impact of metabolic flexibility on organismal performance will likely depend on the costs and benefits associated with changes in both these metabolic traits.

## Data accessibility

Data are available via Dryad (http://dx.doi.org/10.5061/dryad.g0q0q) [15].

## References

[1] Auer SK, Salin K, Rudolf AM, Anderson GJ, Metcalfe NB 2015 The optimal combination of standard metabolic rate and aerobic scope for somatic growth depends on food availability. Funct. Ecol. 29, 479–486. (10.1111/1365-2435.12396)

[2] Killen SS, Glazier DS, Rezende EL, Clark TD, Atkinson D, Willener AST, Halsey LG 2016 Ecological influences and morphological correlates of resting and maximal metabolic rates across teleost fish species. Am. Nat. 187, 592–606. (10.1086/685893) 27104992

[3] Reinhold K 1999 Energetically costly behaviour and the evolution of resting metabolic rate in insects. Funct. Ecol. 13, 217–224. (10.1046/j.1365-2435.1999.00300.x)

[4] Gillooly JF, Brown JH, West GB, Savage VM, Charnov EL 2001 Effects of size and temperature on metabolic rate. Science 293, 2248–2251. (10.1126/science.1061967) 11567137

[5] Hochachka P, Buck L, Doll C, Land S 1996 Unifying theory of hypoxia tolerance: molecular/metabolic defense and rescue mechanisms for surviving oxygen lack. Proc. Natl Acad. Sci. USA 93, 9493–9498. (10.1073/pnas.93.18.9493) 8790358 PMC38456

[6] Norin T, Clark T 2016 Measurement and relevance of maximum metabolic rate in fishes. J. Fish Biol. 88, 122–151. (10.1111/jfb.12796) 26586591

[7] Bennett AF, Ruben JA 1979 Endothermy and activity in vertebrates. Science 206, 649–654. (10.1126/science.493968) 493968

[8] Hayes JP, Garland T 1995 The evolution of endothermy: testing the aerobic capacity model. Evolution 49, 836–847. (10.2307/2410407) 28564873

[9] McKechnie AE 2008 Phenotypic flexibility in basal metabolic rate and the changing view of avian physiological diversity: a review. J. Comp. Phys. B 178, 235–247. (10.1007/s00360-007-0218-8) 17957373

[10] Auer SK, Salin K, Rudolf AM, Anderson GJ, Metcalfe NB 2015 Flexibility in metabolic rate confers a growth advantage under changing food availability. J. Anim. Ecol. 84, 1405–1411. (10.1111/1365-2656.12384) 25939669 PMC4682473

[11] Elliott J 1976 The energetics of feeding, metabolism and growth of brown trout (*Salmo trutta* L.) in relation to body weight, water temperature and ration size. J. Anim. Ecol. 45, 923–948. (10.2307/3590)

[12] Secor SM 2009 Specific dynamic action: a review of the postprandial metabolic response. J. Comp. Phys. B 179, 1–56. (10.1007/s00360-008-0283-7) 18597096

[13] Higgins P, Talbot C 1985 Growth and feeding in juvenile Atlantic salmon (*Salmo salar* L.). In Nutrition and feeding in fish (eds Cowey CB, Mackie AM, Bell JG), pp. 243–263. London, UK: Academic Press.

[14] Rosenfeld J, Van Leeuwen T, Richards J, Allen D 2015 Relationship between growth and standard metabolic rate: measurement artefacts and implications for habitat use and life-history adaptation in salmonids. J. Anim. Ecol. 84, 4–20. (10.1111/1365-2656.12260) 24930825

[15] Auer SK, Salin S, Rudolf AM, Anderson GJ, Metcalfe NB 2016 Data from: Differential effects of food availability on minimum and maximum rates of metabolism. *Dryad Digital Repository*: 10.5061/dryad.g0q0q. PMC5095193 28120798

[16] Armstrong JB, Bond MH 2013 Phenotype flexibility in wild fish: Dolly Varden regulate assimilative capacity to capitalize on annual pulsed subsidies. J. Anim. Ecol. 82, 966–975. (10.1111/1365-2656.12066) 23510107

[17] Weibel ER, Bacigalupe LD, Schmitt B, Hoppeler H 2004 Allometric scaling of maximal metabolic rate in mammals: muscle aerobic capacity as determinant factor. Resp. Phys. Neuro. 140, 115–132. (10.1016/j.resp.2004.01.006) 15134660

[18] McCue MD 2010 Starvation physiology: reviewing the different strategies animals use to survive a common challenge. Comp. Biochem. Physiol. A Mol. Integr. Physiol. 156, 1–18. (10.1016/j.cbpa.2010.01.002) 20060056

[19] Auer SK, Salin K, Anderson GJ, Metcalfe NB 2015 Aerobic scope explains individual variation in feeding capacity. Biol. Lett. 11, 20150793 (10.1098/rsbl.2015.0793) 26556902 PMC4685545

[20] Reidy S, Kerr S, Nelson J 2000 Aerobic and anaerobic swimming performance of individual Atlantic cod. J. Exp. Biol. 203, 347–357. 10607544 10.1242/jeb.203.2.347

[21] Guderley H, Pörtner HO 2010 Metabolic power budgeting and adaptive strategies in zoology: examples from scallops and fish. Can. J. Zool./Rev. Can. Zool. 88, 753–763. (10.1139/Z10-039)

[22] Owen SF 2001 Meeting energy budgets by modulation of behaviour and physiology in the eel (*Anguilla anguilla* L.). *Comp. Biochem. Physiol. A Mol.**Integr. Physiol*. **128**, 629–642. (idoi:10.1016/S1095-6433(00)00340-8)

[23] Alsop D, Wood C 1997 The interactive effects of feeding and exercise on oxygen consumption, swimming performance and protein usage in juvenile rainbow trout (*Oncorhynchus mykiss*). J. Exp. Biol. 200, 2337–2346. 9320259 10.1242/jeb.200.17.2337

[24] Fu S-J, Zeng L-Q, Li X-M, Pang X, Cao Z-D, Peng J-L, Wang Y-X 2009 The behavioural, digestive and metabolic characteristics of fishes with different foraging strategies. J. Exp. Biol. 212, 2296–2302. (10.1242/jeb.027102) 19561220

[25] Millidine KJ, Armstrong JD, Metcalfe NB 2009 Juvenile salmon with high standard metabolic rates have higher energy costs but can process meals faster. Proc. R. Soc. B 276, 2103–2108. (10.1098/rspb.2009.0080) PMC2677234 19324750

