# Correction to ‘Differential effects of food availability on minimum and maximum rates of metabolism’

**DOI:** 10.1098/rsbl.2016.0848

**Published:** 2016-11

**Authors:** Sonya K. Auer, Karine Salin, Agata M. Rudolf, Graeme J. Anderson, Neil B. Metcalfe

*Biol. Lett.* **12**, 20160586. (Published online 18 October 2016) (doi:10.1098/rsbl.2016.0586)

The caption for figure 1 fails to indicate which graph is for which trait, meaning that the figure was unclear. The caption was also erroneously duplicated. The corrected caption is presented here along with the figure.

**Figure 1.**
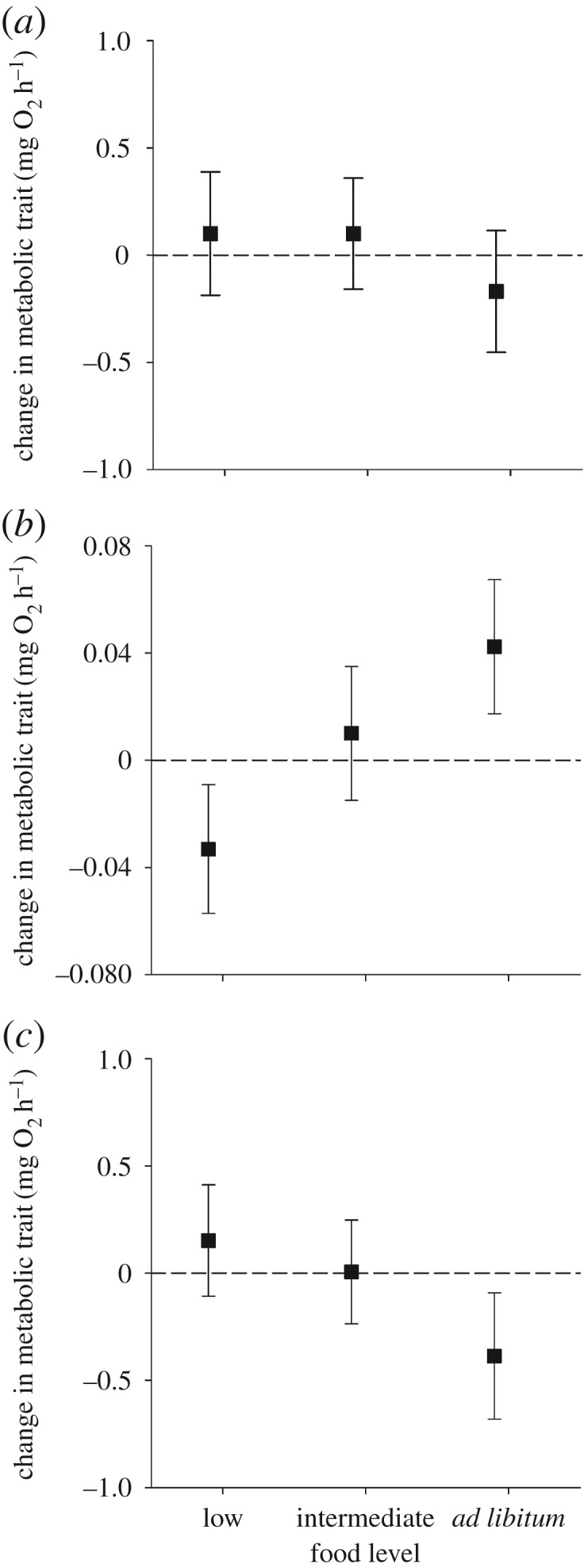
Change in (*a*) maximum metabolic rate (MMR), (*b*) standard metabolic rate (SMR) and (*c*) aerobic scope (AS) of juvenile brown trout as a function of changing food availability. SMR and MMR were first measured after fish had been on an intermediate ration for 28 days and then again after they had been switched to either a lower, intermediate (i.e. the same as previously), or higher ad libitum ration for an additional 28 days. AS is defined as the difference between SMR and MMR for each fish. Plotted are back-transformed metabolic rate values (+95% CI) standardized for a 10 g fish; positive/negative values indicate an increase/decrease in metabolic rate relative to initial values.

